# Bargaining with patriarchy through the life course: obstacles faced (and overcome) by women leaders in Kerala’s health sector

**DOI:** 10.1186/s12939-022-01744-y

**Published:** 2022-10-11

**Authors:** Devaki Nambiar, Gloria Benny, Hari Sankar D

**Affiliations:** 1 The George Institute for Global Health, New Delhi, India; 2 Faculty of Medicine, University of New South Wales, Sydney, Australia; 3 Prasanna School of Public Health, Manipal Academy of Higher Education, Manipal, India

**Keywords:** India, Kerala, Women, Leadership, Challenges, Work-life balance, Gender, Education, Career, Patriarchy

## Abstract

**Background:**

The COVID-19 pandemic has helped shine the spotlight on the role of women’s leadership in tackling the world’s health and health system challenges. The proportion of women occupying senior leadership positions in the health sector is less compared to males, even as they constitute a vast majority of the work force. The South Indian state of Kerala is an exception to this trend, a phenomenon that we sought to understand and contextualise. We undertook a study to understand the personal and professional journeys of some women leaders in the Kerala health sector to determine the antecedents of their leadership positions, the challenges that came their way in leadership, and strategies adopted to overcome these challenges. We also investigated into how these experiences shaped their styles and approaches to leadership.

**Methods:**

We conducted a qualitative study involving semi-structured in-depth interviews with women leaders. Sixteen women leaders were identified from public records and through peer nomination and interviewed in their language of preference following written informed consent procedures. Interviews focused on participants’ professional and personal trajectories, work-life balance, style of leadership, challenges, enablers, lessons learned in their path, and their vision for the health system. The interviews conducted in Malayalam were transliterated into English and thematically analysed using Atlas.Ti8 software by three researchers.

**Results:**

Our study participants were aged 40 to around 80 years, from 8 out of 14 districts of the state. Women leaders in Kerala’s health sector faced challenges through the life-course: during their early school education, in professional service as well as in their roles as leaders. There were myriad experiences – including gender stereotyping and discrimination at the intersection of gender and other social identities. Women developed manifold ways of overcoming them and evolve unique – and again myriad—leadership styles.

**Conclusions:**

Women leaders in Kerala have faced shared challenges through their life-course to climb up the ranks of leadership; each leader has adopted unique ways of overcoming them and developed similarly unique leadership styles. At each life stage there were bargains with patriarchy – involving family members (often as allies), against formal and informal institutional rules, managers, peers and subordinates., which in turn suggests a feminist consciousness on the part of Kerala women leaders as well as the society in which they are seeking to lead.

**Supplementary Information:**

The online version contains supplementary material available at 10.1186/s12939-022-01744-y.

## Introduction

The COVID-19 pandemic has helped train the spotlight on the role of women’s leadership in tackling the world’s health and health system challenges [1]. Women occupy various roles in governance and health service delivery all over the world and have been on the frontline during COVID-19 response, as heads of government and state, health workers, community leaders and in other similar roles [1]. Multiple studies have found that exemplary leadership shown by women leaders has made a great difference during these trying times [2, 3].

Across the globe, women constitute a vast majority of the work force in the health sector, which is comparatively higher when compared to other sectors [4]. The literature also suggests that entry into health and care professions constitutes a form of “respectable femininity” which has been encouraged globally, including in the state of Kerala, where our work is based [5, 6].

In the global context, women have been seen to outnumber men in the frontline cadres; however, this representation dwindles at the level of top leadership positions [7, 8]. Most of the leadership roles are occupied by men in the decision-making structures of the health sector, be it at top global institutions and multilateral organizations, or at government and private sector [9, 10]. Prevalent gender norms, overt gender discrimination and a variety of barriers hinder career progression for women [9]. A systematic review which explored barriers to woman leadership identified gender gaps, lack of career advancement options, stereotyping, and difficulty in achieving work-life balance as barriers by majority of woman leaders in health care [11].

The larger context of patriarchy shapes the ability of persons of all genders to accommodate, negotiate, and perhaps, in rarer instances, challenge norms and expectations in their everyday life [7]. What do such negotiations look like in the context of women’s leadership in the health sector, particularly given that women in health leadership have to arrange, adjust and align their individual interests with decisions that affect a larger population well-being and disease management)?

We sought to explore this question in the context of Kerala, a state in southern India. Kerala is much lauded for its gender empowerment. The presence of women in leadership positions of the district administration and health sector is high when compared to other states [12]. We sought to understand the journeys of some of these women leaders in health to determine the antecedents of their leadership positions, challenges faced and how these were overcome. We were further interested in whether/how these experiences shaped women leaders’ styles and approaches to leadership.

## Methods

### Study setting and design

This study employed qualitative field research methods using in-depth interviews in Kerala. It was nested in a larger five-year collaborative health systems research project supporting health reform processes in the state of Kerala, India. Fieldwork for the study was carried out between June and November of 2019.

### Participant selection

Women leaders were identified in two ways: from public records and through peer nomination. We identified women in senior leadership roles in state and district levels from the health department who are directly in charge of implementing the programs influencing population health. Public health services in Kerala are solely provided by the department of health and family welfare department, Kerala. Thus, our search naturally resulted in reaching out to women employed under state health Department of Health and Family Welfare. The administrative cadre of Kerala’s health department is designed in such a way that, only the individuals with a professional medical qualification could occupy leadership positions starting from the PHC (smallest administrative unit) level to the highest level (Director of Health services). However, during the course of our interviews, we received nominations of women leaders who have contributed significantly to improved community’s health through NGO and community level interventions. The identification was furthered to women leaders working at Local Self Government bodies and then to civil society activists working for health issues. This list of participants was extended to include a traditional healer who had received public acclaim.

The recruitment of participants for the study was carried out using convenient sampling technique. The following inclusion criteria were used to select the participants: women occupying senior administrative roles at state and district level of government health system, and/or nomination from senior functionaries therein, or peer nomination by someone who was pre-selected as a leader. We were also interested in leadership in civil and political society in Kerala.

### Interview process

One member of our research team carried out interviews with the sixteen women leaders in health from Kerala. Potential participants were contacted (via email/phone) and a copy of the participant information sheet, informed consent and interview guide were sent by email. Based on the convenience of participants, in-person interviews were carried out following a detailed written informed consent process, with separate permission for audio-recording. The interviews lasted between 60 and 90 min. The language of each interview was either English, Malayalam, or a combination of the two—based on each participant’s preference.

The interviews focused on participants’ professional and personal trajectories, work-life balance, style and vision of leadership, challenges, enablers, lessons learned in their path, and their vision for the health system. The interview topic guide was drafted based on the scoping review carried out by the team earlier [13–16]. The major themes that were covered through the interview guide included demographic characteristics, challenges faced in leadership role and ways they overcame it. (Interview guide is attached as an Additional file 1). We sought out deeper understanding in how each of the participants had experienced these areas in their personal, professional, family and other societal spaces. So, every thematic area had been further broken down to these levels of engagement.

After each interview was completed, audio-recordings of the English or Malayalam interviews were stored in a password protected database, accessible only to the research team. Field notes were created to support transcripts. Interview audio files were transcribed (and Malayalam sections transliterated) into English transcripts by the second author. English transcripts were sent to the participants for their review. Some gave corrections, including removal of certain portions of the transcript, to assure anonymization. The quality of the transcripts was reviewed in detail by two research team members.

### Analysis

Thematic analysis approach was carried out to analyse the data collected using the Atlas.ti 8 software [17]. A three-member research team was involved in the perusal of data and inductive theme generation. A draft code book was generated using the data from the inductive coding process. Multiple discussions and three major meetings with the team resulted in finalizing the codebook and thematic structure. This included merging some codes with others and devising code families (like work-life balance, challenges, gender norms and discrimination, motivation, leadership). Atlas.ti 8 files from the three team members were merged and analysis were consolidated in a narrative format.

### Ethics considerations

This study was approved by the Institutional Ethics Committee of the George Institute for Global Health (Project Number 05/2019).

## Results

We interviewed sixteen women aged 40 to around 80 years of age (see Table 1), whose area of work spread across 8 out of 14 districts of the state. Out of a total of sixteen participants, eight occupied senior administrative roles at state and district levels. We interviewed five health care providers including senior medical officers, nurses who had been recognized for their nursing sector, and a senior traditional healer. The remaining three participants worked closely with communities in the role of an academic at a teaching hospital, an activist in a prominent health-related Non-Governmental Organisation (NGO), and a Local Self Government (LSG) leader (i.e. *Panchayat*^1^ President).

**Table 1:** Demographic characteristics of the participants

**Age of participants**	
40–50	3
50–60	10
Above 60 years	3
**Education**	
College/Professional education	14
School education	2
**Years of experience**	
10–20	4
21–30	6
> 30	6
**Participants’ employment cadre**	
State and district level administration	8
Healthcare Practitioners (MOs, Nurses, ANMs, Practitioners)	5
Other Leaders (LSG members, NGO representatives, Academics)	3

All the participants we interviewed described challenges through their life course -while seeking education, in professional services, as well as in positions of leadership. Quite often, these involved a trade-off between their professional aspirations and patriarchal norms (related to familial or gender obligations and expectations to which they were held). We detail this in the following sections.

### Education/early career experiences

Most of the women we interviewed had received professional qualifications in medical and social sciences, except for two, who had received basic school level education. They described challenges they faced while acquiring education at different points of time in their lives. To begin with, patriarchal gender norms impacted early school education. One among those two participants had to discontinue her schooling as travelling long distance for acquiring education was considered unsafe for girls.

“The reason for not going (to school) was, school was far away. Those days’ girls going to schools far away by walking were not safe because there were not many classmates with me… There was not anyone to go along with to the school. I had to walk a lot to reach school, need to cross river and all (forest)…I had enthusiasm to study.” (P-09).

Three of our women leaders pointed out the challenge in furthering their education post marriage, as child rearing was a priority for them at that time. In contrast, child rearing responsibilities did not prevent their partners from pursuing higher education.

*“I tried Post graduation during the early phase of my career, that is in 87. I passed out from (anonymized) Medical College and I got married in the same year. And I had my first child in 88, so there was no time for me to go for post graduation that time. Because my husband is also a Doctor and he was doing his post graduation that time when we were married. So that time, naturally what happened was my time went in babysitting and all.” (P-01).*

*“So in 2006 January the course started. By that time it was around 5 years after my course… Then role from one place to another like that… I opted for the PhD program in* < *name of institution* > *. But because my son was small I couldn't join…(as it) was in Delhi.” (P-12).*

A participant who was teaching at a medical college observed that her female students were more inclined to take up non-clinical subjects. She attributed this to their needing (time) to handle child-care duties, in contrast to their husbands (who more often chose clinical subjects, in part because they were not burdened with childcare).

*“…I am teaching student(s) here, especially post-graduate students. They will be coming after MBBS, they will be 22–23 years, that is the time for them to get married and having children… They cannot attend the class regularly, they may have to take long leaves and mainly we have girl students… (for) Community Medicine rarely 1 or 2 boys come. If they compare their professional career and their husbands, I ask them. They are also doctors, they won't take any leave, If the child is sick? These girls have to sit back. They also may be doing PG course. They can also dedicate. What happens is that in the profession, we will be losing.” (P-10).*

### Professional service experiences

Participants reported that executing their professional duties had an impact upon family life, especially when on night duty:

*“In 2007 I requested again for a transfer. Because I had some issues at home in terms of health. Mother in law had a bypass surgery, I had small children. Because of all these issues, I had problem in doing night duty” (P-02).*

*“So they [my family] suffered a lot because I used to go to hospital in between, from 12 midnight to morning 8 o'clock several times. So every alternative day he [husband] would ask 'Oh! Today you are having an emergency call?’” (P-03).*

Participants who worked in government departments noted the common challenge of travelling with difficult transportation options. One participant recounted being full term pregnant and having to take a bus, two jeeps and then another bus to get to work. Two of our participants noted travelling for work came with challenges of security, requiring their partners’ support.

*“In the 'olden times’, my husband used to come to pick me up in the railway station. People used to make fun. You are such a feminist, and you cannot go home alone at 10 o’clock, 12 o’clock. I said No, I have to take my safety into (my own) hands” (P-06).*

The very usage of public transportation to workplace also posed risks of being sexually harassed.

*“…The one I remember is kind of exhibitionism…in the train. One person sitting opposite to me who was completely exposing the relevant part…Usually going and coming back in the same train…I rang the helpline number. They actually responded and when the train came here, there were police around the compartment. By that time he left.” (P-12).*

When asked about experiences of sexual harassment at the workplace, however, our participants noted that they had not experienced this. In response to a different question, one participant mentioned difficulty she faced when having to take her maternity leave. At the time, the official maternity leave entitlement was only three months in total, and because she had taken leave early, she was mandated to return only two months post-partum. Her superior cited lack of human resources as a reason for their insistence that she rejoin. In this context, the formal rules of her profession clashed with her familial aspirations and obligations and then the participant had to approach the senior leadership in the government for sanctioning the leave and work place transfer.

In this instance, the participant’s family was understanding. Yet there were many instances where participants reflected that their families did not understand or could not relate to the situations they confronted at work. In one case, the participant noted that understanding her working life was not even something she could expect from her family; rather it was her role to satisfy the needs and feelings of family members at home.

*“You have to have that sort of professionalism to leave everything there, Come back home and then be a totally different person. I realized it quite late when I would often recount stories, and my daughter one day said, Amma, why do you have to spoil everybody's mood at the dining table… Looking after my husband's feelings and the children's, that was more difficult, because invariably it's a mother who will be blamed…”(P-07).*

### Experiences in leadership roles

Many participants in our study narrated experiences of gender stereotyping while performing duties as supervisors or managers. There were expectations of women’s behaviour as being malleable and being fit for more light or purportedly easier portfolios:

*“Because being a woman, some public, some administrators or some politicians think that we can be influenced by them easily. I have felt it. But that had not been hindrance for my performance. Yet there were incidents where they have thought in that way and behaved to us like that. But we have never surrendered to that” (P-04).*

*“Although there are educated, talented women, there is a glass ceiling always. It’s very difficult for women to break that to a leadership position. And even when women come to leadership positions in a political party or something like that, soft things are given. Like health, social security, all that…So women [are] just doing women's (things).” (P-06).*

A senior traditional healer working in a remote community in the state commented that gender-based discrimination happens in the informal health sector too. She described instances where, even though both spouses were practicing healers, only the men were represented in meetings and professional events.

*“…When men go for a meeting, they never take their wives along. Men come without taking their wife who is involved in it…While I go for the meeting, I don't talk on the stage. Yet they make me sit there. I don't like to talk there. They will talk (on) behalf of me.” (P-14).*

In a similar vein, another participant was offered a transfer on grounds that it would benefit her partner:

“*They came to me with the carrot, if you will take a (district health administration) post and take charge of District health administrator, we will try and bring your husband here.”(P-07).*

Another woman leader said that she felt forced to get a transfer to be in the same location of her husband, who got a position. For another participant however, the idea of transfers as a form of discrimination was not considered, but rather she felt that being posted near each other would reflect gender sensitivity:

*“Only thing I wanted to say is this department should move little more forward to support the women staff. Husband and wife working in one place is not possible, reducing the travel by posting in closer places and things like that.” (P-01).*

There were situations pointed out by study participants where they were discriminated on the grounds of religion and caste. One of the participants while replying to our enquiry about caste-based discrimination in their workplace, reported that.

*“I have heard from others that some of them talk like that behind me, but I have never bothered about it…Some of them have told me that I have never deserved to be [in the current position as she is from a reservation category] and this I heard through others. That is their problem.” (P-16).*

*“…Since I do not have Nair (Hindu- Upper caste surname) at the end of my name, It will be problematic for me, so many of them told me not to go there (State level post)… But I said, that can't be done, I will come and see…Later on, I realized such discrimination [exists] there…It is very silent, not visible though. We can understand that… Then I thought, how we can think like that being this much educated… how ever high the position you are in… the identity of female gender and caste are really existing. Every time that difference is reiterated.” (P- 08).*

### Overcoming challenges through bargaining

Women leaders were often the first to defy patriarchal gender roles in their families and yet, this involved some kind of adjustment or compromise on their part given the predominance of norms related to these roles. In some cases, bargaining meant literally going the extra mile—to attain education and their position. One of our participants recalled,

*“…my mother made me do everything, and I felt it was like me being punished when I was going to school. Early morning, I had to wake up and I had to prepare food for me by myself…wash all my clothes by myself and for my younger siblings also. So I had to do everything at home and have to do it all fast… They didn’t allow me to study after evening 8 pm. So, all that time I got to study was during the day time by adjusting other things.” (P-08).*

Those who were able to progress with their education unequivocally reported the importance of support from their family members. While mentioning about support from their partner, many felt that both the partners being in the same profession is a positive catalyst for maintaining work-life balance.

*“I married a person who is also an activist. And we met at the organizations I worked at previously. So, he perfectly understands what I am doing. And we did not want the children to suffer, so when they were young, we would take turns. When he had to travel, I would stay at home and *vice versa*. Very few times, when both of us were away, our friends pitched in*.” *(P-06).*

*“Support from husband also. I used to reach home at night 8 o clock. My husband also used to come for work (along with me).” (P-15)*

In a sense, therefore, another bargain had to do with the professional pursuits of the partner they would most feasibly be able to have, given their own aspirations. In other instances, participants noted active help given by their partners, meaning that the bargain directly involved defiance of norms by their partners:

*“…he(husband) is always supportive. Most of the time when I couldn’t find an answer for something, he would help me...…He says if you have done something wrong you have to bend down your neck, otherwise walk with your head held high. My daughter has said your profession is your passion. So don't cry….” (P-11).*

*“My husband brought my child to my workplace directly, for me to breastfeed the child. I am so fortunate for that. I had so much support at my house also. My husband and family have never asked me to stay out of a job because I am a woman.” (P-05).*

It was also noted that the support these women received from their co-workers was essential, enabling them to function, and motivating them as leaders.

*“We were able to work only with their (colleagues’) help and support. If we think to do something by ourselves, it may not happen. Help from people and colleagues (is important)…The support they gave us while working with them and thereby the experience was a motivation for the times ahead.” (P-15).*

One participant recounted the lack of mentorship, indicating how she now had stepped in to fill that gap for other members of her family:

*“I had struggled a lot to study; there was no role model or no one to tell me what to study. But it was easy for my sister and my cousins because I could guide them. One is a gynaecologist (now), one is [in] Forensic[s].” (P-16).*

Over their decades of experience, women leaders had designed their own strategies and mechanisms to overcome challenges, be it in their professional or in social or family life. For example, one participant noted that she took complains as a learning experience: *“whatever comes in as a complaint, I never leave it like that. I'll take it and study it properly. That is my thing. Because if somebody is asking, and I always say that they are my best teachers also. If there is no complaint, I will not learn.”* (*P-03).*

Another participant said that she ignored the criticism: *“Things like anonymous letters would be there. We just have to take it in that spirit-like these kinds of people are there in the society. But I have not internalized that. So it was in its own way, that's all” (P-13).*

### Leaders and their dynamic leadership styles

Our participants observed that enduring challenges and going through difficult times in work had shaped their leadership style(s). Having said this, these women leaders approached teamwork and adopted variable leadership approaches – taking responsibility for failures but also trying to motivate colleagues along on pathways to growth:

*“… I am a combination leader. When it is required to take (an) authoritative role, I take that only. But more or less I am a democratic leader. In any disastrous or difficult situation, I take authoritative role. I take authoritative role if the ship we sail is about to fail, I take ownership for the failure. At the same I time, I allow my fellow members to grow along with me and I make sure they get capacity building trainings.” (P-05).*

*“What I used to do is, my style is in two types. I identify within our group who works, I delegate them. Then we don’t need to think about them. But there are some people who are not motivated, so I try to motivate them. When I go to training and all, I try to motivate the team, even though I am not a self-motivated person, I try to motivate others…Always we take opinion, when we have (to) take decisions. I call for programme officers meeting and ask their opinion, but beyond that, the final decision will be, the decided thing has to be carried out.” (P-01).*

Another style we observed was shared, distributed, or collective leadership: *“All the staff in the office are good… If I say something to them, they understand it and do accordingly; they are efficient as they give response quickly. They are not slackers. All of them have to work together to make the system to go forward. Then only I also can move forward.” (P-13).*

In spite of the challenges the women leaders face, they were motivated by the recognition they received from community members, political leaders, as well as superiors and colleagues at work.

*“I received good support from society… ‘we’ achieved these trophies personally for myself and for the PHC… Otherwise also a lot of people, schools, library, District panchayat member… Really society has recognized me…If I am going in a bus, they would take tickets, enquire whether I need food or not.” (P-11).*

*““I got 2012 that social welfare department award, in 2013 State award for best nurse. In 2014, National Florence Nightingale award which I received from Delhi. In 2017, Vanitha Ratnam Puraskar given by our Chief Minister …. If we are really determined to do something we can really achieve it as an individual.” (P-02).*

## Discussion

Our study, while small in scope, revealed the pervasive influence of patriarchy in shaping Kerala women leaders’ experiences of education seeking, professional progression, and leadership – it was indeed throughout the life course! Women did find ways to overcome hardships and to assert their power, nonetheless, with support from their partners, colleagues, and communities (regardless of gender).

Kerala’s high levels of female literacy are well rooted historically in the state’s development trajectory, particularly as compared to other states in India [18–20]. Some have associated educational attainment with empowerment [20]. Indeed, the mere fact of being in leadership roles for many of our participants was associated with being educated. However, participants also noted that gender norms had a significant impact on their ability to receive and advance their education.

Child rearing and care were prioritised by women in their early careers. Participants were expected to opt-out of continuing higher education, in contrast to their spouses. Moreover, spouses in same career did not experience such displacements. This indeed is a finding reflected in many other studies that lay out the tension during the critical period of performing reproductive roles while also career-building and rising to leadership roles [21, 22]. The ‘baby penalty’ borne by women is well documented [23–25], and was also seen in our study.

In addition, we found that female doctors would choose academic subjects on the basis of their ability to accommodate other (family-related) tasks, which were highly gendered, a finding seen in both High Income (US) and Low and Middle Income Country contexts (like Kenya) [25, 26]. That is, if couples chose similar areas of academic focus, men would be exempted from childcare duties, but not women. A narrative review found global evidence suggesting that woman are more interested in teaching than research in academic medicine and among other factors, that ensuring a work-life balance deters them from pursuing a career in research [27]. In a study in Ecuador found that gendered norms influenced their choice of medical specialty even as women may have outnumbered men in the profession overall [28].

Our study found that women working late hours or undertaking night duties impacted their role as caregivers at home. Woman doctors in Pakistan also identified night duty as a major challenge [15]. A study in Ecuador reported how female doctors with nonmedical partners would choose specialties that did not involve night duty [28]. An Indian study focused on night shifts in the health sector reported that women were accomodative of their late working hours because the requirement was relatively infrequent and the fact that they in general had job security and pay [29]. In our study, the challenges of transportation during late working hours was also raised. In general, we found that commuting for work was a major challenge, consistent with findings of other studies with female doctors elsewhere [15]. We found that women had been subjected to sexual harassment even while using public transport to the workplace and this very mode of public transportation posed a danger for women’s safety. This is an experience women working internationally have faced as well [30].

Our study found that various forms of support – logistical (like help with travel) and emotional (like encouragement) – were provided to participants by their spouses when in the same profession. A study carried out in this direction speaks about the enabling feature of ‘dual-physician marriages’ that provides bi-directional understanding of each other emotionally helps to reduce internal conflicts [31]. Women’s leadership based studies also reflect on the importance of spousal and other family member’s support in order to pursue leadership career for women [9, 16, 32]. Evidence suggests that role models and family members have helped motivate women in their careers [16, 33]. Mentoring has been a critical factor in the professional growth of women leaders [14]; in our study, a woman leaders herself stepped into mentorship role for their junior colleagues and future generations. Going forward, as recommended by Javadi and colleagues, there is a need to integrate mentorship into (widened) training opportunities for women that connect students to leaders and build networks [32].

The support of co-workers was mentioned by a participant in our study and has been found elsewhere to be source of support for women leaders [33]. On the other hand, women lacking peer support from peers and supervisors has been found to be a reason for women avoiding leadership roles [16]. This is related to the ‘fear of success’ motive that the literature suggests that the risks and cost of professional advancement are greater than the gains – a phenomenon that does not exist for men [34]. Conversely, in Ethiopia, it was found that public recognition motivated women to seek leadership roles [16], as was the case in our study as well.

The women leaders in our study have faced discrimination based on other intersections of their identity: religion, caste, and geographical location. This has been seen in a 2016 report on barriers to women’s leadership in the United States reports women of colour face difficulty in advancing to leadership positions [35]; while a study in South Africa found that race and gender intersected to create unique experiences of disadvantage and discrimination for black, female managers [36]. Further research on these unique, intersectional challenges, as well as work on strategies to address them are both sorely needed.

The 2021 Global Health 50/50 Gender and Health index assessed the degree to which 201 organisations promote gender equality at the workplace [37]. The report found that only a third of organisations had gender parity in leadership bodies. In our case, while the representation was higher, the glass ceiling would manifest itself in other ways like the pervasiveness of gender stereotyping experiencing a palpable tension between the roles expected of women and of leaders [32, 38, 39]. Even in local self government institutions in Kerala, it has been found that despite wide representation of women as leaders, men have continued to lead actual decision-making [40].

What we found in our study in the health sector has been slightly different. Women leaders in our study were exercising leadership – on their own terms based in variable experiences. Following from this, we did not find a consistent or singular style of leadership among Kerala women leaders: they were people oriented, seeking to enable democratic and collective decision-making, but also would be authoritative when they felt the situation demanded it. There were also women leaders who indicated that their style placed emphasis on communication, relationship building and so on, as is also reported in the literature [14, 32, 41]. Our research suggests that this diversity of experiences, approaches and lack of conformation to a particular mould or form of leadership, may itself be demonstrative of agency on the part of women leaders and reflect the loosening of hegemonic patriarchal norms in the state.

We theorise the combined and variable experiences of our participants as an instantiation of *patriarchal bargain* in the sense proposed by Kandiyoti, involving “the existence of set rules and scripts regulating gender relations, to which both genders accommodate and acquiesce, yet which may nonetheless be contested, redefined, and renegotiated…. women as a rule bargain from a weaker position” [42]. The lifelong experiences of bargaining with patriarchy – during schooling, professional life and in positions of leadership—placed women leaders sometimes in opposition with their family members (for instance when schooling was hindered) and other times in alliance with them (when partners played greater caregiving or supportive roles to support the fulfilment of women’s work). Women experienced lack of mentorship on the one hand, but were also strongly encouraged by recognition from community members, leaders and colleagues. So another bargain was not having that formal or continuous support or recognition, but rather having it in an episodic fashion. We noted that for one participant, being posted to a place of work near one’s spouse was an unsuccessful bargain because this was not a post she wanted. Another participant just assumed that joint postings, while desirable, would not be possible and the bargain would be that women workers be posted close to their partners if possible. Thus, bargains with patriarchy were experienced and interpreted in myriad ways among Kerala women leaders. As Kandiyoti posits, these may have been from a weaker position at the individual level, but taken together, the fact that there has been variation in the challenges faced and perceived, approaches to overcome them, and impact on women’s behaviour and philosophies of leadership, suggests to us forms of agency and the loosening- of patriarchal hegemony – which by design requires conformity of roles, rules and norms. Devika (2019) argues that while the firmament of patriarchy in the state is not dismantled, “the present includes women’s open and veiled struggles against patriarchal control” which are not addressed by the most visible and typical “streams of feminist activism” in the state. She speaks of the struggles of women plantation workers within unions and of women challenging growth model-led ecological predation in the state [43]. There appear to be various such ongoing contestations in the state and as Devika argues, patriarchy in Kerala is not merely a local or even familial phenomenon but rather intertwined closely with women’s roles as workers – and on their labour(s) – both paid and unpaid.

### Limitations of the study

Our sample size was smaller than even we had planned due to logistical and time constraints. While we achieved thematic saturation, we feel a broader sample, including from practitioners in the private sector, would provide important additional insights. We also had a fairly homogenous sample, reflecting some of the biases and gendered realities of the health sector; i.e. those from economically disadvantaged backgrounds including indigenous communities and religious minority groups are less likely to have been represented in our sample. This constrains the generalisability of our findings as well. Another limitation, an ethical dilemma, was posed to us by a senior administrator during recruitment; they cautioned us that focusing only on the work and experiences of certain individuals might demotivate their peers (other potential women leaders working in the health department) who were doing exemplary work but were not yet recognised and thus not recruited in the study. The intention of this work was to understand and increase motivation around women’s leadership; in light of this comment, we have sought not to specifically identify women leaders, but rather anonymously describe their experiences -shared and unique. Going forward, however, the impact of such work on motivation warrants further study and inquiry.

## Conclusion

Women leaders in Kerala’s health sector faced challenges through the life-course: during their early school education, in professional service as well as in their roles as leaders. There were myriad experiences – including gender stereotyping and discrimination at the intersection of gender and other social identities. Women developed manifold ways of overcoming them and evolve unique – and again myriad—leadership styles. We found no single pattern or pathway leading to women’s leadership in our sample, even though it was quite homogenous. We therefore saw various forms of patriarchal bargaining, which in turn suggests a feminist consciousness on the part of Kerala women leaders as well as the society in which they are seeking to lead.

## Supplementary Information

**Additional file 1.** Interview guide. 

## Data Availability

The authors confirm that the data supporting the findings of this study are available within the article.

## Footnotes

1 Panchayat is an institution of local self-government in India given constitutional status through the 73^rd^ Constitutional Amendment of 1992 to devolve decentralized power at the local level. See. https://www.india.gov.in/my-government/constitution-india/amendments/constitution-india-seventy-third-amendment-act-1992
