# Supplementary material for: Bargaining with patriarchy through the life course: obstacles faced (and overcome) by women leaders in Kerala’s health sector
Source: Int J Equity Health. 2022 Oct 11;21:146. doi: 10.1186/s12939-022-01744-y (PMC9552136; doi:10.1186/s12939-022-01744-y)
Supplement: Supplementary file 1 — Additional file 1. Interview guide. [file 12939_2022_1744_MOESM1_ESM.docx]

**Interview guide**

I. Basic information

- Age

- Describe your trajectory from your education onwards (probe: Education;

- Specialization/focus;

- Years of experience;

- Location of experience)

II. Role of women in the health sector

- What is your current role in the Kerala health system?

- How does being a woman affect this role?

- Women are an intersection of different identities (mother, daughter, sister, wife,

artist, scientist, doctor, administrator, confidante etc.)– talk to us about that and

how that has affected your position and contribution to health

III. Work life balance

- How have you balanced the various roles of your life – who has helped you do

this?

- Any experience with common challenges and how they were addressed

o Family obligations hinder career

o Unsupportive employers

o Lack of support for child-bearing

o Limited time with family

o Household duties limit career advancement

o Unsupportive family members (including spouse/partner)

o Experience with mobility – did you have to travel alone? Did you?

IV. Work recognition &amp; Career advancement

- What career obstacles were faced in your career? Describe some incidents

that you recall that really made an impression on you (could be one major

thing or many small things)

- How have you handled these obstacles? Has that changed over time?

- What impact have those challenges had on your way of working now?

- Would you say you have been discriminated against? Were you ever made to

feel inferior, denied opportunities and positions) – why or why not? Describe

- Are you aware of incidents of sexual harassment experienced by you or those

around you? If no, why do you think this has not come up? If yes, how were

they handled (have you experienced unwelcome sexual advances, offensive

remarks, coercion/pressure)

V. General view

- What is your vision or dream about the health system upon how it ought to be helping

the people it serves? (probe – achievement of health equity, anything special for

women?

- What is the role of women health worker in achieving the above?
